# The role of upstream open reading frames in translation regulation in the apicomplexan parasites *Plasmodium falciparum* and *Toxoplasma gondii*

**DOI:** 10.1017/S0031182021000937

**Published:** 2021-09

**Authors:** Chhaminder Kaur, Swati Patankar

**Affiliations:** Department of Biosciences & Bioengineering, Indian Institute of Technology Bombay, Powai, Mumbai 400076, India

**Keywords:** *P. falciparum*, *T. gondii*, translational regulation, upstream ORFs

## Abstract

During their complex life cycles, the Apicomplexan parasites *Plasmodium falciparum* and *Toxoplasma gondii* employ several layers of regulation of their gene expression. One such layer is mediated at the level of translation through upstream open reading frames (uORFs). As uORFs are found in the upstream regions of a majority of transcripts in both the parasites, it is essential that their roles in translational regulation be appreciated to a greater extent. This review provides a comprehensive summary of studies that show uORF-mediated gene regulation in these parasites and highlights examples of clinically and physiologically relevant genes, including *var2csa* in *P. falciparum*, and *ApiAT1* in *T. gondii*, that exhibit uORF-mediated regulation. In addition to these examples, several studies that use bioinformatics, transcriptomics, proteomics and ribosome profiling also indicate the possibility of widespread translational regulation by uORFs. Further analysis of these genome-wide datasets, taking into account uORFs associated with each gene, will reveal novel genes involved in key biological pathways such as cell-cycle progression, stress-response and pathogenicity. The cumulative evidence from studies presented in this review suggests that uORFs will play crucial roles in regulating gene expression during clinical disease caused by these important human pathogens.

## Graphical abstract

**Figure:**
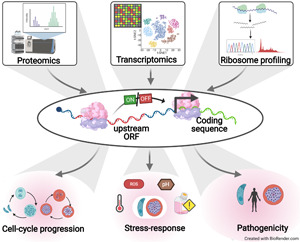

## Introduction

Eukaryotic translation initiation is a tightly regulated, multi-step process that involves scanning of messenger RNA (mRNA) by the preinitiation complex (Kozak, 1980). This complex, comprising of the small ribosomal subunit and numerous initiation factors, scans the mRNA for the start codon (AUG) of the coding sequence (CDS) (Kozak, 1991). The selection of the start codon is governed by the sequence surrounding the AUG codon, i.e. the Kozak sequence, availability of initiation factors, molecules that provide energy and methionyl-tRNAs (reviewed in Hinnebusch, 2011).

Other than these factors, the presence of start codon(s) that lie upstream of the start codon of the main CDS confers another layer of regulation. This is due to the scanning model of translation initiation where the ribosomes recognize the 5′ cap and move along the mRNA towards the 3′ end. During this process, the ribosomes encounter upstream start codons (uAUGs) before the main CDS and therefore, these uAUGs are capable of engaging the ribosome (Kozak, 2002). Similar to uAUGs, upstream open reading frames (uORFs), defined as an upstream start codon followed by an in-frame stop codon, also engage the scanning ribosome with varying capacities, which in turn alters the level of the protein encoded by the main CDS (reviewed in Morris and Geballe, 2000). The presence of these alternative initiation sites constitutes a ‘hurdle’ for the ribosome and usually results in repression of translation of the main CDS. This repression can be relieved by the cellular translation machinery with a multitude of strategies, as and when required (Wang and Rothnagel, 2004; Iacono *et al*., 2005). Hence, uORFs can act as regulatory elements in the 5′ leader sequences of eukaryotic mRNAs. As translation regulation allows the organism to respond more rapidly than transcriptional regulation, uORFs are used by cells to handle a wide range of environmental changes, affecting the survivability of the cell.

The earliest known evidence for uORF involvement in translational control was shown for *Saccharomyces cerevisiae* General Control Non-depressible 4 (ScGCN4), a transcription factor that controls amino acid biosynthesis under conditions of starvation (reviewed in Hinnebusch, 1988). After these early reports, translation regulation by uORFs during stress conditions was shown in numerous organisms including *Homo sapiens*, *Mus musculus*, *Drosophila melanogaster*, *Neurospora crassa*, *Danio rerio*, *Arabidopsis thaliana*, *Zea mays* and higher plants (Iacono *et al*., 2005; Barbosa *et al*., 2013; Chew *et al*., 2013; von Arnim *et al*., 2014; Lei *et al*., 2015; Young and Wek, 2016; Zhang *et al*., 2018, 2019; Chen and Tarn, 2019; Silva *et al*., 2019; Wu *et al*., 2019).

### Indicators of uORF-mediated gene regulation

**Fig. 1.**
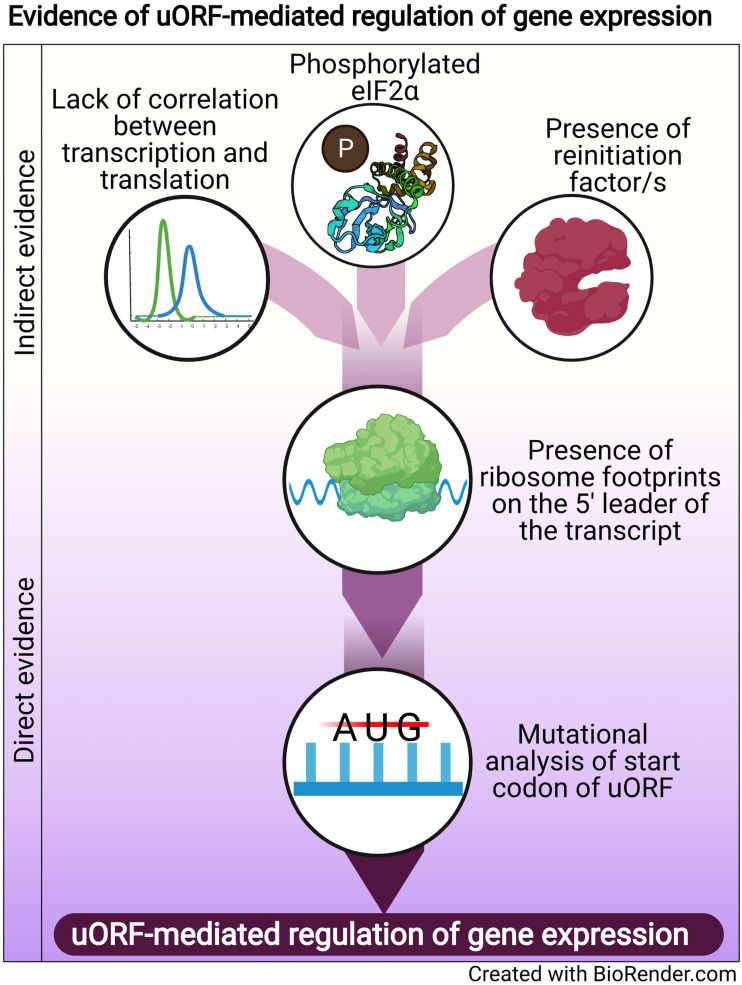
Evidence of uORF-mediated gene regulation

Regulation of translation is one mode of post-transcriptional gene regulation (PTGR). The presence of PTGR in cells can be inferred by several key cellular features. One such feature is a lack of correlation between the peak mRNA level and the protein abundance for a given gene and/or temporal delay between the transcription and the translation of the gene (de Sousa Abreu *et al*., 2009; Maier *et al*., 2009; Liu *et al*., 2016). These can be explained by multiple factors such as stability of mRNA, the secondary structure of the transcript, post-translational modifications and *cis*-regulatory elements including uORFs (Araujo *et al*., 2012; Carpenter *et al*., 2014). Therefore, while studying the regulation of a gene by uORFs, a delay between the transcription and translation of the gene is often used as a preliminary indicator of translational control (Fig. 1).

Other indirect indicators that point towards uORF-mediated gene regulation are the proteins/factors that the translation machinery uses to overcome the challenge posed by the uORF. To cope with uORF-mediated translation repression, and allow protein synthesis to occur from the CDS, a cell adopts unconventional mechanisms of translation: reinitiation, leaky scanning, ribosome shunting, and the use of internal ribosome entry sites (IRES) (reviewed in Morris and Geballe, 2000; Skabkin *et al*., 2013; Silva *et al*., 2019). In brief, reinitiation is a phenomenon where the ribosome, rather than dissociating after termination at the stop codon of a uORF, successfully re-initiates translation at the start codon of the main CDS. On the other hand, leaky scanning occurs when the ribosome scans past and skips the start codon of the uORF, consequently initiating translation at the start codon of the main CDS (reviewed in Silva *et al*., 2019). Even though other unconventional translation mechanisms, such as ribosome shunting and IRES, have been observed in eukaryotes, they are more prevalent in viruses (Yang and Wang, 2019) and hence, the discussion here will be limited to reinitiation and leaky scanning. Factors that promote reinitiation and leaky scanning are indicators of a dampening of global translation and upregulation of certain genes. For some genes that are controlled by reinitiation/leaky scanning, there is an involvement of uORFs in regulating gene expression, particularly during a cellular stress response. Therefore, an indirect indicator of uORF-mediated translational control can be the upregulation and/or modification of factors that regulate either reinitiation by employing reinitiation factors, or leaky scanning by phosphorylating the eukaryotic initiation factor 2 *α* (eIF2*α*) (Fig. 1).

The phosphorylation status of eIF2*α* was demonstrated for the first time in the case of the GCN4 transcript, in which the translation of the CDS is regulated by uORFs. The choice of translating the CDS rather than the uORFs is driven by phosphorylation of eIF2*α* (reviewed in Hinnebusch, 2005). Similarly, during the integrated stress response (ISR) in *Saccharomyces cerevisiae*, phosphorylated eIF2*α* promotes translation of transcripts required for handling the stressor *via* reinitiation (Dever *et al*., 1992; Lu *et al*., 2004). Similar mechanisms that involve relieving uORF-mediated repression by phosphorylated eIF2*α* have been discovered for numerous genes (Vattem and Wek, 2004; Dang Do *et al*., 2009; Zhao *et al*., 2010; Palam *et al*., 2011; Baird *et al*., 2014; Zach *et al*., 2014; Aktas *et al*., 2015; Cnop *et al*., 2017; Guan *et al*., 2017; Asano, 2021). The phosphorylation of eIF2*α*, which is carried out by members of the eIF2*α* kinase family (Pakos-Zebrucka *et al*., 2016; Wek, 2018; Costa-Mattioli and Walter, 2020), leads to global inhibition of protein synthesis and preferential translation of transcripts encoding proteins involved in mediating the adaptive response. These studies indicate that the phosphorylation status of eIF2*α* is a global indicator for translational regulation of large numbers of genes, some of which could be controlled by uORFs.

A more definitive role for uORFs in translational regulation is provided by the presence of ribosomal footprints on the 5′ leader of the transcripts undergoing PTGR (Schneider-Poetsch *et al*., 2010; Garreau de Loubresse *et al*., 2014). This provides a snapshot of the dynamics of translation on each transcript by determining the positions of the ribosomes engaged in elongating an ORF (Brar *et al*., 2012; Ingolia *et al*., 2014). Such studies in yeast and humans revealed that uORFs are the major contributors of ribosome occupancy in the 5′ leaders of transcripts (Calvo *et al*., 2009; Brar *et al*., 2012; Ingolia *et al*., 2014; Johnstone *et al*., 2016), suggesting that the presence of ribosome footprints in the 5′ leader of the transcript is a distinctive feature that indicates PTGR *via* uORFs. Ribosome footprints along the entire length of certain transcripts show that when the upstream regions are loaded with ribosomes, the CDS has lower ribosome occupancy (Ingolia *et al*., 2014). These data reinforce the notion that the presence of uORFs stalls the ribosome before it can reach the main CDS, resulting in repression of CDS translation.

Direct evidence of uORFs regulating the translation of a particular transcript is provided when mutation of the start codon of the uORF results in a loss of repression/regulation of the gene (Harigai *et al*., 1996; Reynolds *et al*., 1996; Ruan *et al*., 1996; Sarrazin *et al*., 2000; Schlüter *et al*., 2000; Diba *et al*., 2001; Kwon *et al*., 2001; Jousse *et al*., 2001; Warnakulasuriyarachchi *et al*., 2003; Zhang and Dietrich, 2005; Lee *et al*., 2007; Song *et al*., 2007; Calvo *et al*., 2009; Devlin *et al*., 2010; Spevak *et al*., 2010; Qiao *et al*., 2011; Armata *et al*., 2013; Bancells and Deitsch, 2013; Tennen *et al*., 2013; Capell *et al*., 2014; Wu *et al*., 2014; Kumar *et al*., 2015; Guerrero-González *et al*., 2016). A summary of direct and indirect evidence indicating the involvement of uORFs in mediating gene expression regulation is shown in Fig. 1.

### Translational regulation mediated via uORFs in apicomplexan parasites *Plasmodium falciparum* and *Toxoplasma gondii*

Apicomplexans belong to a large phylum of parasitic alveolates and due to their complex life cycles involving multiple hosts including humans, some members of the phylum cause the widespread occurrence of diseases. For example, malaria and toxoplasmosis are caused by *P. falciparum* and *T. gondii*, respectively (Sabin and Olitsky, 1937; Jacobs, 1963). These parasites exhibit many developmental stages in different hosts and therefore, must regulate the expression of their genes in a highly coordinated fashion for survival and transmission to complete their life cycles. Gene expression is regulated at multiple levels, including transcription and translation (White *et al*., 2014; Vembar *et al*., 2014, 2015, 2016; Holmes *et al*., 2017; Bennink and Pradel, 2019; Hollin and Le Roch, 2020; Sharma *et al*., 2020).

There is evidence for uORFs playing substantive roles in translational control in apicomplexan parasites; this evidence includes high frequencies and widespread distribution of uORFs among large numbers of transcripts (Bunnik *et al*., 2013; Caro *et al*., 2014; Kumar *et al*., 2015; Srinivas *et al*., 2016; Hassan *et al*., 2017; Holmes *et al*., 2019; Markus *et al*., 2021). Additionally, ribosome profiling studies in *P. falciparum* and *T. gondii* parasites reveal footprints in the 5′ leader sequences of transcripts (Lacsina *et al*., 2011; Bunnik *et al*., 2013; Caro *et al*., 2014; Hassan *et al*., 2017; Holmes *et al*., 2019). Recent discoveries of clinically important genes, such as *var2csa* in *P. falciparum* (Lavstsen *et al*., 2003; Salanti *et al*., 2003, 2004; Amulic *et al*., 2009; Bancells and Deitsch, 2013) and ApiAT1 in *T. gondii* (Rajendran *et al*., doi: 10.1101/798967, in consideration), that are regulated translationally by uORFs further reinforce the impact of these small, yet important features in translational regulation of gene expression. In the next sections, the current status of the field will be summarized and the need to further understand the phenomenon of uORF-mediated PTGR in apicomplexan parasites will be highlighted in detail.

## Upstream ORFS in *Plasmodium falciparum*

### A long uORF regulates translation of the *var2csa* gene

The first example of uORF-mediated translational regulation in *P. falciparum* was shown for a gene implicated in pregnancy-associated malaria (PAM), also termed malaria in pregnancy: *var2csa* (Lavstsen *et al*., 2003; Salanti *et al*., 2003, 2004; Amulic *et al*., 2009; Bancells and Deitsch, 2013). This gene is a variant of the *var* gene family in *P. falciparum* that consists of ~60 *var* genes encoding erythrocyte membrane protein 1 (PfEMP1). These proteins help the parasite evade clearance by the spleen of the host by binding to the endothelial lining of blood vessels (Kraemer and Smith, 2006). The *var* gene family has also been implicated in cerebral malaria, one of the major symptoms of severe malaria caused by *P. falciparum* that results due to sequestration of infected RBCs to capillaries in the brain (reviewed in van der Heyde *et al*., 2006). This sequestration is due to the binding of PfEMP1 proteins to receptors such as CD36, thrombospondin and intercellular adhesion molecule 1 found on the surface of different cell types (Baruch *et al*., 1996; Smith *et al*., 2000, 2013; Rowe *et al*., 2009).

The transcription profile of members of this gene family is unusual, with only one of the *var* genes expressed at a given time (Scherf *et al*., 1998) and a switch of gene expression between different *var* genes occurring at the rate of 0.03–2% (Gatton *et al*., 2003). The mechanisms that regulate the switch are complex and regulation occurs at various levels including sub-nuclear organization, epigenetic regulation, *cis*-acting DNA elements, transcriptional regulation as well as translational repression (reviewed in Deitsch and Dzikowski, 2017). Due to the importance of the *var* gene family in multiple clinical manifestations of severe malaria caused by *P. falciparum*, understanding the expression of the members of this gene family has been of intense interest.

**Fig. 2.**
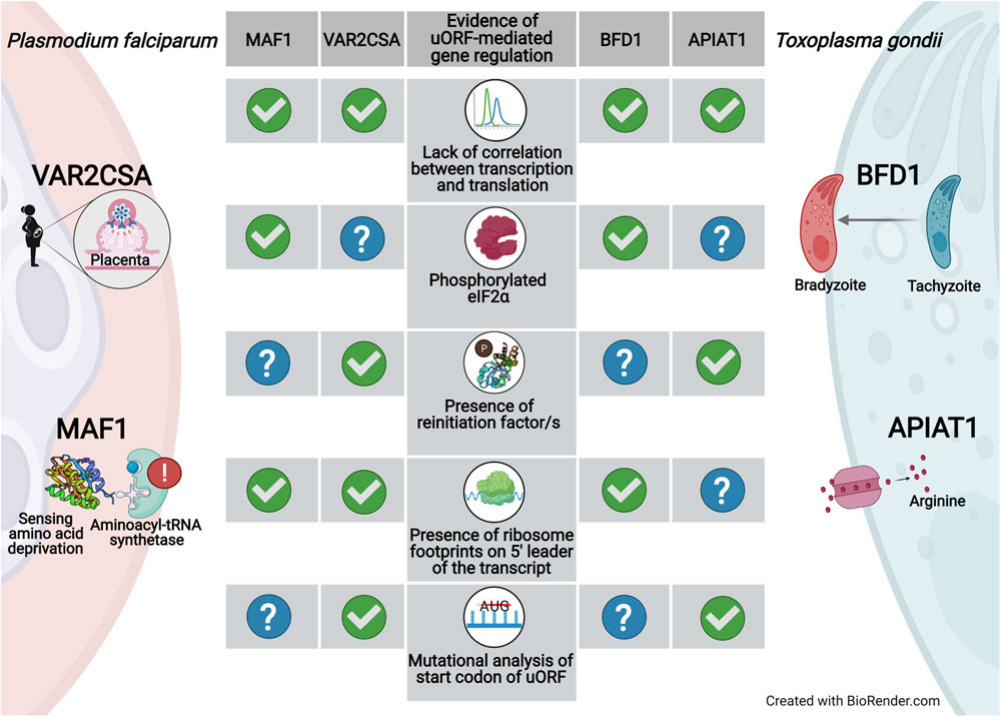
Examples of genes in *P. falciparum* and *T. gondii* that are regulated *via* uORFs

One variant of this gene family that has attracted clinical attention is *var2csa*. Parasites expressing *var2csa* adhere to chondroitin sulphate A (CSA) found in the placenta of pregnant women and block the supply of oxygen and nutrition to the fetus, thereby resulting in PAM (Salanti *et al*., 2003, 2004). The VAR2CSA protein is observed in parasites obtained from the placenta of pregnant women and also in parasites that are selected for adherence to CSA in lab cultures (Salanti *et al*., 2003, 2004; Mok *et al*., 2008). However, the *var2csa* transcripts are also observed in parasites that do not adhere to CSA in lab-grown cultures, indicating that these transcripts are not translated (Mok *et al*., 2008; Chan *et al*., 2017). This lack of correlation between transcription and translation is a clear indicator of PTGR (Fig. 2), which is regulated by a 360 nucleotide-long uORF in the 5′ leader of the transcript (Dzikowski *et al*., 2007; Amulic *et al*., 2009; Bancells and Deitsch, 2013).

In a series of direct experiments that included mutational analysis of the start codon of the uORF, this 360 nucleotide-long uORF was shown to cause translational repression of the *var2csa* transcript. Hence, it was of interest to understand how this repression is relieved to express the VAR2CSA protein when required. In cultured parasites, detailed molecular analysis showed that this switch depends on reinitiation of the *var2csa* transcript after the uORF is translated (Bancells and Deitsch, 2013). In parasites derived from placental samples, this reinitiation was shown to occur due to the presence of *Plasmodium* translation enhancing factor (PTEF). PTEF is highly upregulated in parasites sequestered in the placenta and appears to bind to ribosomes to allow efficient reinitiation of translation at the *var2csa* CDS (Chan *et al*., 2017). A homologue of this reinitiation factor is found in *P. reichenowi*, the chimpanzee malaria parasite having an AT-rich genome similar to *P. falciparum* (Otto *et al*., 2014), indicating that this protein may be conserved in closely related parasites or may be required for handling translation of transcripts generated from AT-rich parasite genomes.

The molecular factors that lead to the expression of PTEF in a CSA-rich environment are still unknown and further studies need to be undertaken to understand the structure and the interacting partners of PTEF in the asexual stages to provide further clarity regarding its role. These detailed studies are particularly important, as recent reports have shown that the serum of non-pregnant individuals (men and children) contain antibodies recognizing the VAR2CSA protein (reviewed in Gnidehou and Yanow, 2021). While the authors discuss technical issues such as cross-reactivity to other proteins that cannot be ruled out, they also mention that deregulation of the uORF-mediated repression of the *var2csa* gene might play a role in these clinical findings.

### The high prevalence of uORFs in the *P. falciparum* genome leads to repression of translation

Reports establishing translational regulation of the *var2csa* gene led to an interest in understanding whether this phenomenon was observed in other genes as well. Subsequent studies provided indirect evidence that regulation by uORFs could be more prevalent in *P. falciparum* than previously anticipated. For example, the *P. falciparum* transcriptome displays widespread occurrence of uORFs (Caro *et al*., 2014), with 99% of the transcripts containing at least one uORF in their 5′ leader (Kumar *et al*., 2015). This number is extremely high when compared to human transcripts where only 49% of the transcripts contain at least one uORF in their 5′ leader (Calvo *et al*., 2009). With a prediction of an average of 11 uORFs per CDS (Kaur *et al*., 2020), *P. falciparum* exhibits the highest ever recorded number of uORFs in a transcript.

The high frequencies of uORFs are a reflection of the distinctive genome of *P. falciparum* whose composition is skewed heavily towards adenine (A) and thymine (T) nucleotides (Gardner *et al*., 2002). This leads to a high probability of finding AT-rich start and stop codons in the 5′ leader sequence, thereby giving rise to uAUGs and uORFs. These large numbers of uAUGs and uORFs pose a significant challenge to the parasite's cytoplasmic translation machinery, resulting in repression of the downstream gene. This was shown by the insertion of a short sequence, containing several uORFs, upstream of a reporter that resulted in almost complete loss of reporter activity. When all the start codons of the uORFs in this inserted sequence were mutated, reporter activity was regained, showing that the repression was at the level of translation (Kumar *et al*., 2015).

Each uORF has a very different ability to engage the scanning ribosome and experimental approaches have been used to study the features that contribute to the repressive capacity of uORFs. Such features are the Kozak sequence, codon composition, length of the uORF, and the distance between the uORF and the CDS (Kaur *et al*., 2020). Further, bioinformatics was used to predict translatability of an ORF (therefore, the repressiveness of the uORF) by calculating the probability of translation initiation and elongation of ORFs in the *P. falciparum* genome (Srinivas *et al*., 2016). The model utilizes positional features comprising of the Kozak sequence and compositional features comprising of the codon topography of the ORF to predict translation initiation and elongation probabilities of the ORF, respectively.

The notion of translation repression by certain uORFs is reinforced by the presence of ribosome footprints on the 5′ leaders of transcripts expressed in the intra-erythrocytic asexual stages of *P. falciparum*, supporting the hypothesis that these large numbers of uORFs can engage ribosomes. Ribosome profiling studies found a rampant occurrence of ribosome density associated with 5′ leaders of transcripts compared to the 3′ UTRs (Bunnik *et al*., 2013; Caro *et al*., 2014). The data suggest the active translation of uORFs present in the 5′ leader sequence, with some instances where transcripts showed a higher abundance of ribosome footprints on the 5′ leader than on the CDS, leading to low translational efficiency of the CDS (Caro *et al*., 2014). Interestingly, only 50% of the ribosome footprints overlapped with the predicted uORF (Caro *et al*., 2014), pointing towards the presence of non-canonical translation initiation sites in *P. falciparum*. If indeed non-canonical translation initiation sites are widespread, the current predictions of the numbers of uORFs may be a gross underestimate. As the repressive capability of different uORFs has now been assessed by experimental/bioinformatics analysis and ribosome profiling, a holistic analysis of all these genome-wide datasets would shed light on specific uORFs regulating the expression of classes of genes. Based on the evidence that uORF-mediated regulation is a strategy employed to handle stress responses, such classes of genes might be physiologically relevant in host–pathogen interactions and establishing the pathogenicity of this parasite.

Another indicator of translational control of gene expression, a delay between the peak of transcript abundance and translation of those transcripts, has also been observed in *P. falciparum*. Early reports of a cascade of gene expression in synchrony with the asexual life cycle stages (Bozdech *et al*., 2003; Foth *et al*., 2011) suggested that transcription occurs only when the protein is required. Subsequent studies showed an absence of correlation between the peaks of transcripts and their encoded protein products for ~30% of the genes (Le Roch *et al*., 2004; Bunnik *et al*., 2013), indicative of PTGR for these genes. It is noteworthy that the *var* gene family that is under multiple forms of regulation has ~5 times more uAUGs and uORFs than other genes (Kumar *et al*., 2015).

Translation repression of the downstream CDS in the presence of uORFs can be alleviated by unconventional translation mechanisms: reinitiation and/or other mechanisms including leaky scanning. The role of reinitiation in the translation of VAR2CSA during PAM has been discussed in the previous section. However, the use of non-canonical translation mechanisms to circumvent translation repression caused by the uORFs is not limited to this gene. Indeed, it was demonstrated that reinitiation occurs in the case of the hsp70 transcript in the presence of a native uORF and synthetic uORFs suggesting that there is a widespread occurrence of reinitiation in the asexual stages of *P. falciparum* (Kaur *et al*., 2020).

More and more evidence points towards uORFs playing roles in translational regulation during the asexual stages of the intra-erythrocytic developmental cycle (IDC). As the asexual stages of *P. falciparum* are the cause of the clinical symptoms of malaria, a better understanding of uORF-mediated translational regulation may lead to the identification of new targets for therapeutic interventions.

### Upstream ORFs in stress conditions

The role of uORFs in the stress response in yeast and mammals is well studied (Hinnebusch, 2005; Silva *et al*., 2019; Houston *et al*., 2020). However, this area of research requires more focus on *P. falciparum*, more so because of the widespread occurrence of uORFs. During its complicated life cycle, *P. falciparum* faces a variety of external conditions that are hostile to the parasite. As is the case with other parasites, *P. falciparum* has also evolved to use complex strategies to adapt to the changing environment (Camus *et al*., 1995). While the shift of host from mosquito to human is one of the major challenges faced by the parasite due to drastic differences in the two hosts’ biology, understanding how the parasite responds to various stress conditions that it faces in the human erythrocytes holds importance from the clinical perspective of malaria treatment.

During the IDC, *P. falciparum* experiences a periodic rise in temperature every 48 h due to the host inflammatory response (Brown, 1912). The temperature during these febrile episodes can elevate to 40–41°C (Kwiatkowski, 1989). The adaptive response to the cyclical heat stress experienced by intra-erythrocytic parasites has been studied at the level of the transcriptome (Oakley *et al*., 2007; Rawat *et al*., doi: 10.1101/752543, in consideration). However, as translational responses afford a rapid adaptation mechanism, it would be informative to study whether uORFs play a role in heat stress by checking the phosphorylation status of PfeIF2*α* and differential ribosome occupancy during this stress condition.

Another stress faced by *P. falciparum* during its intra-erythrocytic cycle is the lack of essential amino acids, especially isoleucine. This stress arises from the fact that inside the red blood cell, the parasite salvages amino acids by degrading haemoglobin (Francis *et al*., 1997). However, of the 20 amino acids, isoleucine is completely absent in the *α* and *β* chains of haemoglobin (Sherman, 1977). Therefore, the parasite depends on an exogenous supply of isoleucine through the plasma of the host (Liu *et al*., 2006). Since isoleucine is an essential amino acid, the human host also depends on external sources of isoleucine to survive (Soeters *et al*., 2004) and in situations of malnourishment, isoleucine pools in the human host can drop significantly (Baertl *et al*., 1974).

Lack of an exogenous supply of isoleucine can lead to a delayed-growth phenotype, where the parasites enter a dormant state as a response (Babbitt *et al*., 2012). This response has been linked to phosphorylation of PfeIF2*α via* PfeIK1, an orthologue of GCN2 that is responsible for phosphorylation of eIF2*α* under nutrient starvation conditions in yeast (Hinnebusch, 2005; Fennell *et al*., 2009; Babbitt *et al*., 2012). A possible role of uORFs in the translation of the transcripts required for adaptive response to this nutritional stress faced by *P. falciparum* can be illustrated by identifying transcripts having differential ribosome occupancy in parasites that are deprived of isoleucine. Further, ribosome profiling of PfeIK1 knock-out parasites would also reveal classes of genes that are under regulation by uORFs.

There is preliminary evidence to support the notion that translational regulation mediated by uORFs occurs during isoleucine starvation stress. The Maf1 protein (a repressor of RNA polymerase III) is a part of the target of rapamycin complex 1 (TORC1) pathway that responds to stress caused by nutrient deprivation in *S. cerevisiae* and mammals (Loewith and Hall, 2011). Maf1 represses transcription of highly abundant tRNAs and ribosomal RNAs through its function as a regulator of RNA polymerase III (Upadhya *et al*., 2002; Boguta, 2013; Moir and Willis, 2015). In nutrient-rich conditions, Maf1 remains inactive due to phosphorylation (Pluta *et al*., 2001; Shor *et al*., 2010), while under starvation conditions, it is de-phosphorylated and the activated protein binds and inhibits RNA polymerase III (Vannini *et al*., 2010). Although the majority of proteins involved in the TORC1 pathway have been lost in the *Plasmodium* genus during genome reduction, an orthologue of Maf1 has been identified in *P. falciparum* (Serfontein *et al*., 2010; McLean and Jacobs-Lorena, 2017). Ribosome profiling data show a significant presence of ribosome footprints on the 5′ leader sequence of the Maf1 transcript indicating that the Maf1 CDS is poorly translated despite being transcribed in all stages of IDC (Caro *et al*., 2014). Mutant parasites that have a disrupted 5′ leader sequence of Maf1 fail to recover from a state of dormancy induced due to isoleucine starvation (McLean and Jacobs-Lorena, 2017). This points towards the role of uORF-mediated regulation of Maf1 translation during this nutritional stress response. This phenomenon warrants further investigation (Fig. 2).

Another physiologically important stressor is treatment with the antimalarial drugs chloroquine and artemisinin since they constitute a source of oxidative damage to the parasite by inducing free radical production (Pandey *et al*., 2001; Haynes and Krishna, 2004; Zhang *et al*., 2010). Parasites treated with dihydroartemisinin, a derivative of artemisinin showed enhanced phosphorylation of eIF2*α*, a key regulator of stress adaptation (Zhang *et al*., 2017), suggesting a possible role of PTGR in overcoming the drug-induced stress. Increased cases of resistance to antimalarial drugs suggest that parasites have evolved to enhance their adaptive response to drug-induced stress, thus decreasing drug susceptibility (Rocamora *et al*., 2018). This has been shown in the case of artemisinin, where increased levels of phosphorylated PfeIF2*α* induce latency in parasites, thereby causing them to re-emerge later when the drug pressure has subsided (Zhang *et al*., 2017). These studies could be extended by identifying transcripts that have repressive uORFs due to enhanced ribosome occupancy.

Clearly, there are gaps in our understanding of the adaptation responses mounted by *P. falciparum* during these stress conditions. Filling in these gaps by studying the role of uORFs in stress responses would be necessary to gain deeper insights into parasite biology, especially in conditions of clinical relevance.

## Upstream ORFS in *Toxoplasma gondii*

### Translation regulation of the arginine transporter TgApiAT1 by uORFs

The first direct evidence of uORF-mediated translational regulation was shown for an arginine transporter protein (TgApiAT1) that is involved in the uptake of arginine in *T. gondii* (Rajendran *et al*., doi: 10.1101/798967, in consideration).

*Toxoplasma gondii* depends on nutrients derived from its host (Coppens, 2014; Zuzarte-Luís and Mota, 2018) and a nutrient for which the parasite is auxotrophic is the amino acid arginine (Fox *et al*., 2004). Hence, there is a dedicated plasma membrane transporter (TgApiAT1) for the uptake of arginine (Rajendran *et al*., 2017). Depletion of this amino acid results in the formation of bradyzoites, the latent forms of this parasite that form tissue cysts (Fox *et al*., 2004; Butcher *et al*., 2011). To maintain the virulent tachyzoite stage and cause infection, parasites need to sense the availability of arginine and respond accordingly to maintain the intracellular levels of arginine by regulating the expression of the transporter TgApiAT1.

The arginine-dependent expression of TgApiAT1 is mediated *via* an upstream ORF present in the 5′ leader sequence of the transcript (Rajendran *et al*., doi: 10.1101/798967, in consideration) (Fig. 2). The uORF codes for a conserved peptide that is hypothesized to function in a similar manner to the arginine attenuator peptide found in *S. cerevisiae* (ScAAP) (Rajendran *et al*., doi: 10.1101/798967, in consideration). The ScAAP stalls the ribosome and prevents it from reaching the downstream CDS in arginine-rich condition (Spevak *et al*., 2010). Conversely, in arginine scarcity, ribosomes can reach and translate the downstream CDS (Wei *et al*., 2012; Wu *et al*., 2012). A similar switch is used by *T. gondii* for modulating the TgApiAT1-dependent uptake of arginine in varying arginine conditions (Rajendran *et al*., doi: 10.1101/798967, in consideration). Given the extensive occurrence of uORFs in *T. gondii*, we believe that this might be among the first of many studies that unravel the existence of uORF-mediated translational regulation.

### Ribosome profiling in *T. gondii* points towards widespread translational regulation by uORFs

Indirect evidence for the role of uORFs in the translational regulation of many genes can be found in *T. gondii*. Similar to *P. falciparum*, the transcripts of *T. gondii* also have a widespread occurrence of uORFs. At least one uORF has been predicted in 90% of transcripts with annotated 5′ leader sequences (Markus *et al*., 2021). This number is 1.8 times higher than the reported number in human transcripts where only 49% of the transcripts contain at least one uORF in their 5′ leader sequence (Calvo *et al*., 2009). Evidence of translation occurring in the 5′ leaders of transcripts in *T. gondii* has been provided in two recent ribosome profiling studies that demonstrate a high prevalence of ribosome footprints on the long 5′ leaders of transcripts (Hassan *et al*., 2017; Holmes *et al*., 2019). Ribosomal occupancy on uORFs is indicative of the fact that ribosomes are engaged in translating uORFs rather than the CDS, thereby exerting translational control over the expression of the gene.

In an attempt to study translational control of genes that provide an adaptive advantage to the stress posed by the extracellular environment, comparative ribosome profiling of extracellular and intracellular tachyzoites was performed. This study identified more than a thousand transcripts that vary at the level of ribosome occupancy in intracellular and extracellular parasites, implying there is a possible widespread usage of translational regulation to cope with the stress imposed by the extracellular environment on *T. gondii*. However, a bioinformatics analysis of the sequences 10 nucleotides upstream and downstream of the translation initiation sites of uORFs and CDS yielded scores that were indicative of unfavourable translation initiation at uORFs. After further bioinformatics analysis, the authors concluded that mRNA secondary structures are more likely to regulate translation efficiency in *T. gondii* (Hassan *et al*., 2017). Nonetheless, for stress responses in *T. gondii*, it would be useful to further study the transcripts with more favourable scores for the translation initiation sites of uORFs as compared to their downstream CDS.

### Upstream ORFs play a crucial role in the development of latent cysts in *T. gondii*

*Toxoplasma gondii* tachyzoites develop into bradyzoites under certain conditions (reviewed in Cerutti *et al*., 2020). Bradyzoites are the latent stage of *T. gondii* that persist as tissue cysts and cause reinfection when the immune system of the host lapses (Dubey, 1998; Montoya and Liesenfeld, 2004). While the host immune response can lead to stress that initiates bradyzoite formation *in vivo* (Bohne *et al*., 1993; Lüder *et al*., 1999), conversion of tachyzoites to bradyzoites *in vitro* can be induced under various stress conditions, such as pH change, heat shock, nutritional stress, stress to the endoplasmic reticulum, mitochondrial inhibition, presence of nitric oxide, signalling through secondary messengers such as cAMP, and other *in vivo* factors (Bohne *et al*., 1993; Soete *et al*., 1993; Weiss *et al*., 1995, 1998; Dubey, 1998; Kirkman *et al*., 2001; Fox *et al*., 2004; Narasimhan *et al*., 2008). Stage conversion that can be triggered by a multitude of external stressors is highly reminiscent of an ISR that is controlled by uORFs in other eukaryotes (reviewed in Young and Wek, 2016).

Another indicator of translational regulation, possibly through uORFs, is phosphorylation of eIF2*α* which has also been reported for bradyzoite conversion. TgIF2*α* is phosphorylated during alkaline stress when the developmental shift from tachyzoite to bradyzoite occurs (Sullivan *et al*., 2004; Narasimhan *et al*., 2008). Disruption of this phosphorylation by either deleting TgIF2KB (Augusto *et al*., 2021) or inhibiting TgIF2KA (Augusto *et al*., 2018), both kinases responsible for phosphorylating TgIF2*α*, leads to significant loss of stage conversion.

The molecular factor responsible for the stage conversion was unidentified until the recent discovery of a master regulator, the bradyzoite formation deficient 1 (BFD1) protein that encodes a transcription factor, which triggers the conversion of tachyzoites to the latent tissue cyst form (Waldman *et al*., 2020). Stress-dependent expression of BFD1 appears to be regulated at the translational level because although the transcript is detected both in tachyzoites and in bradyzoites (a marginal 1.5- to 3.6-fold upregulation in bradyzoites), the protein is expressed only in bradyzoites (Waldman *et al*., 2020) (Fig. 2).

As bradyzoites can be formed in culture by a variety of stressors and their stage conversion coincides with the phosphorylation of TgIF2*α*, it would not be far-fetched to infer that uORFs play a role in the process. Most satisfyingly, evidence for the involvement of uORFs in translational regulation was provided by the observation that parasites expressing BFD1 without its 5′ leader can differentiate into bradyzoites even in the absence of any stress. This strongly alludes to the presence of regulatory *cis*-acting elements in the 5′ leader that act as a switch to turn on gene expression under stress conditions. The translational switch of the gene has been hypothesized to be under the control of four uORFs present in its 2.7 kb-long 5′ leader sequence (Waldman *et al*., 2020).

Understanding the control of BFD1 gene expression will have crucial implications from a clinical perspective, as it would allow the development of drugs that inhibit the conversion of tachyzoites to persistent bradyzoites. Bradyzoites cannot be eradicated by any clinically approved drugs; however, the number of parasites that transition from tachyzoites to bradyzoites can be suppressed by the small molecules tanshinone IIA and hydroxyzine (Murata *et al*., 2017). Treatment with another compound, guanabenz displays reduced formation of brain cysts in mouse models (Benmerzouga *et al*., 2015). Furthermore, the compound prevents the conversion of latent cysts to tachyzoites by inhibiting the de-phosphorylation of eIF2*α*, thereby thwarting the parasite's attempt to cause reinfection (Konrad *et al*., 2013). It would be possible to develop such drugs if light could be shed on the molecular mechanism that controls the switch to bradyzoite formation.

## Concluding remarks

Given the sheer number of uORFs and wide prevalence of ribosomal footprints on the 5′ leader sequences in the Apicomplexan parasites, *P. falciparum* and *T. gondii*, their role in mediating translational regulation is certainly under-recognized. Efforts to understand translational regulation in these parasites is gradually gaining momentum (reviewed in Rao *et al*., 2017), and in this review, we highlight selected examples of genes that are regulated by uORFs giving rise to clinically relevant pathophysiology in the life cycles of these parasites. Due to the requirement of novel translation factors that promote non-canonical strategies of handling the ‘hurdles’ created by uORFs, such as reinitiation and leaky scanning, further research in this area may lead to the identification of parasite-specific, essential proteins that might serve as drug targets for therapeutics. We conclude by predicting that, with transcriptome, proteome, ribosome profiling and bioinformatics analyses giving genome-wide pointers towards genes and pathways that might be subjected to uORF-mediated PTGR, the role of uORFs in regulating translation will surely be an area of intense research in the future.
